# Association of Periodontal Disease with Activity of Crohn’s Disease

**DOI:** 10.3390/medicina59122154

**Published:** 2023-12-12

**Authors:** Miloš Živić, Nebojša Zdravković, Bojan Stojanović, Bojan Milošević, Željko Todorović, Miljan Adamović, Nataša Zdravković

**Affiliations:** 1 Department of Dentistry, Faculty of Medical Sciences, University of Kragujevac, 34000 Kragujevac, Serbia; milos.zivic@medf.kg.ac.rs; 2 Department of Medical Statistics and Informatics, Faculty of Medical Sciences, University of Kragujevac, 34000 Kragujevac, Serbia; 3 Department of Surgery, Faculty of Medical Sciences, University of Kragujevac, 34000 Kragujevac, Serbia; bojan.stojanovic01@gmail.com (B.S.);; 4 Clinic of Surgery, University Clinical Center Kragujevac, 34000 Kragujevac, Serbia; 5 Department of Internal Medicine, Faculty of Medical Sciences, University of Kragujevac, 34000 Kragujevac, Serbia; natasasilvester@gmail.com; 6 Clinic of Hematology, University Clinical Center Kragujevac, 34000 Kragujevac, Serbia; 7 Pharmacy Institution “Zdravlje Lek”, 11000 Belgrade, Serbia; zdravljelek@gmail.com; 8 Clinic of Gastroenterohepatology, University Clinical Center Kragujevac, 34000 Kragujevac, Serbia

**Keywords:** Crohn’s disease, periodontal disease, Crohn’s disease activity indexes, periodontal indexes

## Abstract

Introduction: Crohn’s disease (CD) is a chronic inflammatory granulomatous disease that can affect the entire gastrointestinal tract. It is characterized by various extraintestinal manifestations (EIMs), of which oral manifestations (OMs) are often possible. One of the possible OMs is periodontal disease (PD), a chronic inflammatory condition of the supporting tissues of the teeth. This study aimed to show the existence of a mutual relationship between the clinical activity of PD and the clinical and endoscopic activity of CD. Materials and methods: One clinical and two endoscopic indexes were used for the assessment of CD activity and clinical attachment loss (CAL), bleeding on probing (BOP), pocket probing depth (PPD), and radiographic bone loss (RBL) in a dental panoramic tomogram to assess PD in CD patients. Results: A total of 38 patients underwent the entire study process, of which 20 patients had CD and 18 patients had CD and PD. Considering all CD activity scores, there were 26 patients with active disease; half of them had PD, and 85.7% of operated patients had active CD. The values of CAL, PPD, BOP, and RBL were higher in active CD patients than those in remission, except for BOP when comparing to the CDAI score, which was higher in those in remission of CD. Conclusion: The results of this study indicate that there is a connection between the activity of CD and worse conditions of the supporting tissues of the gums in the oral cavity, so it is important to keep in mind the necessity of referring patients with CD to a dentist for timely and adequate therapeutic measures.

## 1. Introduction

Inflammatory bowel diseases (IBDs) comprise two entities, Crohn’s disease (CD) and ulcerative colitis (UC). They represent chronic inflammation of the gastrointestinal tract, which can affect the entire GIT in the case of Crohn’s disease. CD and UC are idiopathic IBDs characterized by periods of relapse and remission. Inflammation is dominantly present in the lining of the colon (sometimes also in the small intestine) in patients with UC. In patients with CD, inflammation, which is mainly segmental and transmural, can be registered in all parts of the digestive tract (from the oral cavity to the anus), especially in the small and large intestine [1,2]. Numerous extraintestinal manifestations of these diseases can also occur. IBD is clinically characterized by recurrent inflammation of gastrointestinal tract segments and diverse clinical manifestations, which are often very unpredictable and sometimes fatal [3,4].

Until now, it is known that the intestinal microbiota plays an important role in the development and maintenance of IBD, although the specific effect of all microorganisms that can affect it has been insufficiently investigated [5]. Several genes are associated with an increased risk of developing CD. So far, the most studied are NOD2 and IL23R, whose mutations are found in about 20% and 10% of people with CD. Also, mutations in the genes ATG16L1, IRGM, and CARD15 predispose patients to the manifestation of CD. Although the mechanism of all the genes that can be affected has not been sufficiently elucidated so far, their identification enables a better understanding of the disease and helps in the development of new diagnostic and treatment strategies [6,7,8].

Research indicates that the prevalence of IBD worldwide is increasing, but the necessity of hospital treatment is statistically significantly higher in patients with CD [5,9]. The frequency of CD is higher in developed countries and urban areas than in rural areas and developing countries. The first symptoms of CD usually appear in the second to fourth decade of life. Previous research has shown that there is no significant difference in the occurrence of CD by gender [1]. There is an increased incidence of CD in the first or second generation among populations migrating from low-incidence regions to high-incidence regions. This information points to the importance of environmental and early exposure to factors influencing the development of CD [1,5,9].

Periodontal disease (PD) is a chronic inflammatory condition of the supporting tissues of the teeth. It is caused by bacteria that form a sticky biofilm on the teeth. In the earlier stage, it is characterized by gingivitis, and clinically, redness and swelling of the gums, which bleed easily when irritated, are observed. If left untreated, it can progress to periodontitis, when the periodontal ligament and alveolar bone are affected. It is a more severe form of PD characterized by the appearance of periodontal pockets and causes receding gums, terminal loosening, and the loss of teeth [10].

Factors that are important for the development of periodontitis are diet (mainly carbohydrates), bad habits, diabetes, family history, certain medications, and immunodeficiency. Diagnosis is made based on a visual and tactile examination of the gingival tissue, probing, and radiographic evaluation to determine the presence and assess the depth of periodontal pockets [11].

There is evidence that the risk of CD is increased in people with a history of PD, suggesting that systemic inflammation in people with PD may trigger or exacerbate current IBDs [12]. Intestinal bacterial flora, immune-mediated inflammatory processes, and certain risk factors are thought to play a common role in both PD and CD. The pathogenesis and whether the interaction between these diseases is one-way or two-way is still not fully explained [12,13].

This study aimed to show the existence of a mutual relationship between the clinical activity of PD and the clinical and endoscopic activity of CD using indexes for the assessment of disease activity and to assess the prevalence of PD in patients with CD.

## 2. Patients and Methods

This study was approved by the Ethics Committee of the Faculty of Medical Sciences in Kragujevac, University of Kragujevac, and the Ethics Committee of the University Clinical Center Kragujevac. 

### 2.1. Patients

The inclusion criterion for this study was de novo diagnosed Crohn’s disease based on an endoscopic examination of the colon and pathohistological findings of a biopsy taken during the endoscopic examination. The exclusion criteria were patients who were under the age of 18, pregnant or nursing, had limited legal responsibility or reduced cognitive abilities, had chronic diseases, or had used therapy or procedures that may affect the examination of parameters in the two weeks before or at the time of the research or were edentulous. 

### 2.2. Assessment of Crohn’s Disease Activity

The clinical activity of Crohn’s disease was determined using the Crohn’s Disease Activity Index (CDAI) [14], the Harvey Bradshaw index [15], and the Van Hess index [16]. These indices include data on the number of daily stools, the presence of abdominal pain, the subjective condition of patients, the existence of extraintestinal manifestations, the use of probiotics and opioids, the body mass index of patients, and the values of blood parameters.

The clinical activity of a disease is the assessment of the disease at a given point in time. It is important to select induction therapy and evaluate the need for hospitalization, or the efficacy of a drug. Clinical classification categorizes a disease as mild, moderate, or severe depending on the following factors: response to therapy, presence of malnutrition, dehydration, systemic toxicity, presence of abdominal tenderness, mass or obstruction, degree of weight loss, and anemia. Symptoms do not necessarily correlate with objective assessments of disease activity, such as endoscopy, cross-sectional imaging (cecum), or biomarkers (C-reactive protein (CRP) or fecal calprotectin), leucocytes, and hemoglobin.

Endoscopic activity was assessed using the Crohn’s Disease Endoscopic Severity Index (CDEIS) [17] and the Simple Endoscopic Score for Crohn’s Disease (SES-CD) [18]. Endoscopic activity was determined based on the existence, characteristics, and extent of inflammation and ulceration in the digestive tract by segment.

Also, the localization of CD depending on age and the predominant type of CD was assessed using the Vienna and Montreal classifications.

### 2.3. Assessment of Periodontal Disease

The presence of periodontitis was assessed using the following periodontological indexes: the clinical attachment level (CAL) [19], bleeding on probing (BOP) [20], and the periodontal pocket depth (PPD) [19,21]. The CAL represents the distance from the enamel–cementum border to the bottom of the gingival sulcus or periodontal pocket; BOP informs us if there is inflammation and bleeding; and the PPD represents the distance from gingival margins to the bottom of the gingival sulcus/periodontal pocket. All of these classifications are presented in Table 1. 

**Figure 1:**
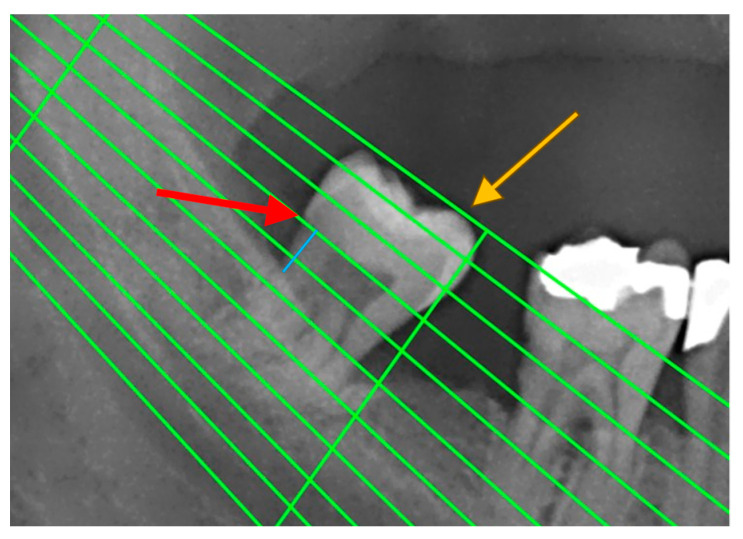
An example of measuring radiographic bone loss using a Schei ruler, where the red arrow indicates the line tangent to the cementoenamel junction, the yellow arrow indicates the line tangent to the highest point of the crown, and the blue line represents approximately 1.5 fields between the cementoenamel junction and the alveolar bone level.

Periodontal disease was also confirmed by measuring radiographic bone loss (RBL) on dental panoramic tomograms (DPTs). All DPTs were taken using an Orthophos XG 3D device (Sirona Dental Systems GmbH, Bensheim, Germany) in the Department of Dentistry at the Faculty of Medical Sciences, University of Kragujevac. To clarify alveolar bone levels and root apices of the entire dentition, all radiographic images were enhanced in the picture editing software GIMP (GNU Image Manipulation Program, Software version 2.10) and were subsequently printed and used for measuring the distance [22] (Figure 1). Periodontal disease staging was assessed from the classification of 2018 [19,23] with extracted referent values by stages (Table 1).

### 2.4. Statistics 

The Statistical Package for Social Sciences software (SPSS, version 27, Chicago, IL, USA) was used for statistical processing and data analysis. Variables were described using frequency distribution for categorical variables and means and standard deviation for continuous variables, as well as medians and the interquartile range, depending on the normality test. After the normality test, the Mann–Whitney U Test and independent samples *t*-test were used accordingly for scale variables. A Chi-square test was used for categorical variables and statistical significance; Fisher’s exact test, continuity correction, and Pearson Chi-square testing were used where needed. Sensitivity and specificity are shown using receiver operating characteristic (ROC) curves and areas under the ROC curves (AUCs).

## 3. Results

This study aimed to examine the correlation between the activity indexes of CD and PD and to assess the prevalence of PD in patients with CD. A total of 38 patients underwent the entire study process, of which 20 patients had CD and 18 patients had CD and PD. The clinical and demographic characteristics are shown in Table 2.

The mean value of age of all 38 patients was 39.45 ± 13.29 years. The duration of Crohn’s disease varied from one month to 25 years depending on the patient. The mean value was 7.05 ± 6.4 years, and there was no statistical significance comparing groups with PD and without PD. The clinical score (CDAI) and endoscopic score (CDEIS and SES-CD) mean values for all patients were 115.11 ± 71.86, 4.18 ± 5.2, and 6.92 ± 8.67, respectively. Of all patients, 12 (31.58%) had clinically active CD, considering their CDAI score, six with PD and six without PD. Using the CDEIS and SES-CD scores, there were 26 (68.4%) and 19 (50%) patients with active CD, respectively. There was no statistically significant difference between the groups. Considering all CD activity scores, there were 26 patients with active disease, and half of them had PD. The BMI mean value difference was statistically significant between CD patients with and without PD (Table 2).

All patients that underwent surgery (36.84%) belonged to either group i0 (57%) or group i1 (43%) of the Rutgeerts score, representing that there were no lesions or less than five aphthous lesions after surgery, respectively. It is important to emphasize that the difference in the mean values of the disease duration in patients with PD who underwent surgery compared to those who did not was clinically significant, and the values were 9.43 ± 7.73 and 5.67 ± 5.16, respectively. Also, 85.7% of operated patients had active CD.

The results of our study, using the Vienna and Montreal classifications, showed that in more than half (60%) of the patients with Crohn’s disease, the disease was localized in the ileocolon (group L3). In most patients, 44.7% had inflammatory-type CD and 34.2% had stricture-type CD. The penetrating type was represented in 21.1% of cases, of which all had perianal complications. In the CD+PD group, 22% of patients had perianal complications, and in the CD group, that percentage was 15%. Also, the ileum and colon were mostly affected in both groups, 66% and 50% in CD+PD and CD, respectively.

**Figure 2:**
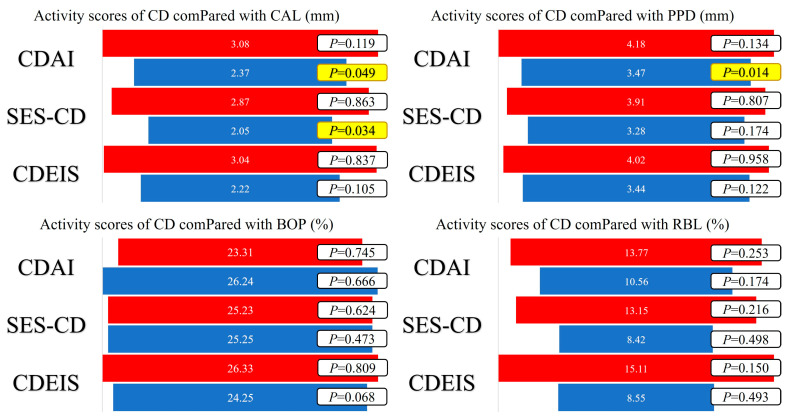
Average values of CAL, PPD, BOP, and RBL according to CD activity scores and statistical significance of the obtained values using the independent sample *t*-test (red—active disease; blue—remission; yellow—statistical significance (*p* < 0.05)).

The periodontal disease index mean values were calculated for BOP—25.24 ± 11.44 (%), CAL—2.61 ± 0.96 (mm), and PPD—3.71 ± 1.09 (mm). Presented by stage of periodontal disease, Table 3 shows the statistically significant differences between PD stages for the CD activity scores (CDEIS and SES-CD), as well as for PD activity parameters (CAL and PPD). The mean value of RBL in all patients was 11.66 ± 12.87 (%), with significantly higher values in the CD + PD group (23%). The average values of CAL, PPD, BOP, and RBL according to the CD activity scores are presented in Figure 2.

**Figure 3:**
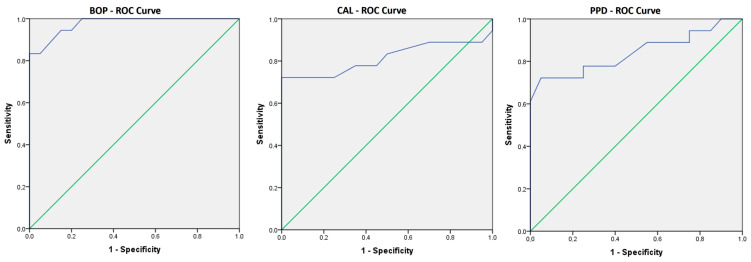
ROC curves showing the sensitivity and the specificity of BOP, CAL, and PPD in prediction of oral manifestations. These ROC curves show that all three are good predictors of belonging to the group that developed oral manifestations.

By using the ROC curve to assess the predictive ability for the presence of OM in CD patients, we used the CAL, PPD, and BOP values, and it was shown that BOP values can be an indicator of the occurrence of OM in CD patients (AUC = 0.976, *p* < 0.001) with a sensitivity of 94.4%, while the specificity is 85% and the limit value is 23.00. Also, the values of CAL (AUC = 0.814, *p* = 0.001) and PPD (AUC = 0.840, *p* < 0.001) can be indicators as well, with similar sensitivities of 72.2%, and 77.8% and specificities of 100% and 75%, respectively. The threshold value of the CAL was 2.90; for PPD, this was 3.30. Statistically significant sensitivities and specificities are shown using ROC curves in Figure 3. There were no significant differences in the values of CRP, fecal calprotectin, leucocytes, and hemoglobin between CD patients with and without PD.

## 4. Discussion

It is known that in the active state of CD, there are significantly more inflammatory processes in the body, and the same is the case with PD and intestinal flora imbalance [1,2,24]. To point out the importance of the correlation between the activity of CD and PD, using the mentioned methods for determining both the activity and the severity of the disease, the importance and need for further research in this area was pointed out. The results of this study indicate that there is a connection between the activity of CD and worse conditions of the supporting tissues of the gums in the oral cavity. The prevalence of CD patients with PD in our study was 47.4%. The study by Lauritano et al. shows that the prevalence of any oral manifestation (OM) in patients with CD varies from 0.7% to 37% [25], and Lankarani et al. reported in their study that the prevalence rate of extraintestinal manifestations ranges from 6 to 47% [26]. In a study by Alvarado Julio et al., the presence of OM was reported in as many as 63% of patients with IBD, the most common of which is gingivitis in as many as 55% of cases [3].

Large differences in the frequency of the disease reported in different studies may be due to different study designs, study objectives, the number of subjects in each study, and the inexperience of the researchers or doctors in diagnosing or conducting the research. It is important to keep in mind the lack of a universal classification of OMs or their severity in people with Crohn’s disease, as well as the difficulty in comparing the parameters used in different studies.

Oral manifestations that can occur in patients with CD are divided into specific ones in the form of cobblestone mucosa, granulomatous cheilitis, and seals on the mucosa, and non-specific ones in the form of aphthae, fissures, other types of cheilitis, lichen planus, periodontal disease, dental caries, and others. They are, in most cases, asymptomatic, and the prevalence of these OMs is 0.5% and 37% [27], while according to some earlier studies, it ranged from 12.7 to 21% [25]. Generally, the most common OM in IBD is aphthous ulcerations, which are clinically hard to distinguish from aphthous ulcerations in healthy people, so a biopsy and/or Anti-Saccharomyces cerevisiae antibody (ASCA) test is necessary [28].

Our results are in agreement with a cohort study that evaluated the association between IBD and periodontitis. The authors concluded that patients with IBD in whom the disease is active have a more severe clinical picture and extent of periodontitis compared to those in remission [29]. In our study, although there were the same number of patients with active disease in both the CD+PD and CD groups, the CD activity clinical score values were higher in the CD+PD group, which represents a third of the group. Also, a study by Zhang et al. showed a higher risk of PD in patients with IBD [30].

Following the Vienna and Montreal classifications, the results indicate that the CD+PD group has more patients with perianal complications than the CD group, which may be clinically significant, although no statistically significant difference was obtained. The reason for this may be the greater spread of the disease, since the disease in the CD+PD group involved more patients with perianal complications than in more parts of the gastrointestinal tract.

Using the new classification from 2018, it is difficult to compare the results obtained in studies carried out before the establishment of this classification [31].

Our results showed that the BOP values were statistically significant in the group of patients with CD with OMs compared to the CD group. The mean value of BOP we found was 25.24%, which is similar to some other studies [32,33,34,35], but higher values are also reported [36,37]. In a study by Vavricka et al., the results of BOP were shown, which are more pronounced in patients with CD [38], which is also shown in the study of Imai et al. [39]. This does not correlate with the study in which the results indicate that the presence of dental plaque was associated with a lower risk of CD [40], and the values of BOP were similar in the IBD and control groups. Patients with CD had higher PPD and CAL values in one study [35] compared to the control group, which is contrary to the study where they found higher PPD values in the control group compared to the IBD patients [34]. Although the PPD values were higher in the control group, higher CAL values were observed in more places in the oral cavity in patients with IBD in both mentioned studies, which may be the reason for the immediate remission of PD and a previous greater loss of periodontal tissue. It is important to note that, in several studies, the clinically significant information is that the measured values of CAL were >6 mm, even in up to 32% of cases, although no statistically significant differences between groups were obtained, both for CAL and for BOP and PPD [32]. Our results show significantly higher CAL values in 47% of patients with a mean value greater than 3 mm, while in some studies where periodontitis was present, the CAL value was less than 3 mm (mean: 0.9 mm) [35]. In most studies, there is information about a significantly greater number of missing teeth in IBD patients compared to control groups [29,32,37,41]. The study by Baima et al. found a significantly higher prevalence of PD in IBD patients compared to the control group. It has been shown that stage III and stage IV disease are significantly more common in IBD patients. The difference in PPD (≥5 mm) and CAL values is also significantly higher in IBD patients. What is important is that this study showed the same significance using the 2018 classification used in our study [31,42]. Statistical significance was shown by comparing the presence of periodontal disease and the age of the subjects, where subjects with PD had a higher mean age. We can see such data in other studies, where it has been shown that patients with CD aged 51–65 years have significantly more severe forms of PD as well as higher values of CAL and PPD [42].

So far, we know that there is an association of pathogenesis between IBD and PD, and that both diseases may depend on genetic, environmental, microbial, and immunological factors. In the study by Tanwar et al., patients with IBD showed a significantly higher number of OMs compared to the control group, which is explained by difficult oral hygiene and a changed diet due to stomach problems, which causes a more suitable environment in the oral cavity for the development of microorganisms and the progression of periodontitis [43]. In addition to studies indicating more frequent OMs in CD, there is also a study in which no statistical or clinical significance of the frequency or severity of the clinical picture of PD was shown [34].

The duration of IBD is a clinically significant factor both for determining the quality of life in patients and for prognostic therapeutic planning. It may also be significant for explaining certain results obtained in studies comparing clinical and endoscopic IBD scores with other parameters, as in this study. Our results show that the duration of CD (7.05 ± 6.4 years) is shorter than those values in another study [42], where the importance of disease duration and surgery related to IBD is also indicated, which, in that study, are negatively associated with periodontitis. Based on this, it can be concluded that the timely and regular control of IBD can play a protective role, both for the underlying disease and for the onset and progression of oral manifestations. Our results indicate a significant difference in the number of operated patients, and this is higher in the CD group without OM, with statistically significantly more cases with a type-i1 Rutgeerts score (Table 2). It is important to state that, in our study, the illness duration was significantly higher in patients who underwent surgery.

## 5. Conclusions

In conclusion, our data show that active CD causes increased bone loss that is visible in orthopantomograms and causes more inflammatory processes in the oral cavity, which is most often manifested in the form of gingivitis.

The shortcoming of this study is possibly the lack of a number of patients with a wider demographic coverage, as well as a larger number of measured variables, which is certainly planned in the continuation of this research. The strength of this study is reflected in the fact that several parameters of CD and PD activity were compared with the Vienna and Montreal classifications, the Rutgeerts classification, and with some values from serum, which can be significant in the objectivity of determining the scores and finding the correlation of all variables with PD stages using new classification.

Collaboration between dentists, gastroenterologists, immunologists, and infectious disease specialists, along with other healthcare professionals, is necessary to provide holistic oral–systemic healthcare. Optimal dental care should reduce the intestinal supply of pathogenic oral bacteria, offering innovative methods to reduce the risk and severity of IBD. It is important to keep in mind the necessity of referring patients with CD to a dentist for timely and adequate therapeutic measures to stabilize the disease, and thus significantly reduce inflammatory processes in the body.

## Figures and Tables

**Table 1:** Referent values by stages of periodontal disease (classification of 2018).

Stage	Clinical Attachment Loss (CAL)	Radiographic Bone Loss (RBL)	Bleeding on Probing (BOP)	Probing Pocket Depth (PPD)
Healthy	0–0.9 mm	None	0–9%	0–2.9 mm
I	1–2.9 mm	<15%	<10%	3–3.9 mm
II	3–3.9 mm	15–33%	11–30%	4–4.9 mm
III	4–4.9 mm	33–50%	31–50%	5–5.9 mm
IV	≥5 mm	>50%	>50%	≥6 mm

**Table 2:** Clinical and demographic characteristics of the subjects presented as “mean value ± standard deviation” or “median (The Interquartile Range—IQR)” depending on the normality test.

		CD + PD Group (n = 18)	CD Group (n = 20)	*p*-Value
Age (years)		42.50 (18.00)	31.00 (22.50)	0.021
Illness duration (years)		7.50 (6.75)	3.50 (11.25)	0.212
Gender	Male (n)	10	11	
	Female (n)	8	9	
BMI		26.42 ± 5.66	22.59 ± 4.21	0.026
CDAI score		115.44 ± 75.06	114.80 ± 70.81	0.978
CDEIS score		3.00 (8.25)	2.00 (2.75)	0.383
SES-CD score		6.00 (9.75)	3.00 (4.00)	0.328
Vienna and Montreal classifications *		Class 3 in 72.22%	Class 3 in 50%	0.484
Surgery (%)		27.78	45	/
Rutgeerts		i0-22.2% i1-5.5%	i0-20% i1-25%	/
BOP (%)		34.61 ± 8.61	16.80 ± 5.49	0.000
PPD (mm)		4.42 ± 1.11	3.07 ± 0.56	0.000
CAL (mm)		3.15 ± 1.10	2.12 ± 0.40	0.001
RBL (%)		22.00 (11.25)	1.00 (2.00)	0.000
Fecal calprotectin (ug/g)		115.35 (934.20)	104.60 (225.20)	0.808
Hemoglobin (g/L)		138.50 ± 25.55	133.05 ± 18.94	0.457
CRP (mg/L)		4.45 (14.20)	1.60 (7.65)	0.149
Leucocytes (10^9^/L)		7.69 ± 3.68	7.44 ± 2.07	0.802

* Representing localization of Crohn’s disease—class 3 is the small intestine. Orange colored cells represent statistically significant *p*-values.

**Table 3:** Median and IQR of activity scores of CD and PD compared to stages of periodontal disease and statistical significance shown using * Kruskal–Wallis test.

CD and PD Activity Parameters	Stages (Number of Patients)				
	Stage I (n = 5)	Stage II (n = 9)	Stage III (n = 3)	Stage IV (n = 1)	*p* Value *
CDAI (Median (IQR))	41 (74)	137 (130.5)	181 (/)	/	0.062
CDEIS (Median (IQR))	0 (3)	2 (4.5)	14 (/)	/	0.017
SES-CD (Median (IQR))	0 (6)	3 (4.5)	21 (/)	/	0.016
BOP (Median (IQR))	40 (15)	30 (12.5)	35 (/)	/	0.332
CAL (Median (IQR))	1.9 (1)	3.2 (0.45)	4.2 (/)	/	0.001
RBL (Median (IQR))	20 (8.5)	23 (10)	16 (/)	/	0.618
PPD (Median (IQR))	3.1 (0.6)	4.7 (0.55)	5.7 (/)	/	0.001

Orange colored cells represent statistically significant *p*-values.

## Data Availability

The authors consent to the publication of this study, including their data contained within the article.
